# High prevalence of low vitamin D status in the Czech Republic: a retrospective study of 119,925 participants

**DOI:** 10.1038/s41430-025-01587-0

**Published:** 2025-03-03

**Authors:** Drahomira Holmannova, Pavel Borsky, Jan Kremlacek, Jan Krejsek, Lenka Hodacova, Anabela Cizkova, Zdenek Fiala, Lenka Borska

**Affiliations:** 1 Department of Preventive Medicine, Faculty of Medicine in Hradec Kralove, Charles University, 500 03 Hradec Kralove, Czech Republic; 2 Department of Medical Biophysics, Faculty of Medicine in Hradec Kralove, Charles University, 500 03 Hradec Kralove, Czech Republic; 3 Department of Clinical Immunology and Allergology, University Hospital Hradec Kralove and Faculty of Medicine in Hradec Kralove, Charles University, 500 03 Hradec Kralove, Czech Republic; 4 Synlab czech s.r.o., 186 00 Praha, Czech Republic

**Keywords:** Risk factors, Biomarkers, Malnutrition, Nutritional supplements

## Abstract

**Objectives:**

Given the high worldwide prevalence of vitamin D deficiency and its role in numerous diseases affecting mortality and morbidity, this study seeks to determine the prevalence of low 25-hydroxyvitamin D levels in the Czech Republic, where population-level data are currently lacking.

**Study design:**

This retrospective study utilized a large dataset to analyze 25-hydroxyvitamin D levels over an extended period.

**Methods:**

We analyzed data from 119,925 individuals aged 0–100 years categorizing them as sufficient (75–250 nmol/L), insufficient (50–75 nmol/L), or deficient (<50 nmol/L). We also examined levels of CRP, homocysteine, and their correlations with 25-hydroxyvitamin D levels across age groups. Age, sex, sampling month, sunlight exposure (monthly and annual), and influenza virus positivity were assessed for their relationship with the 25-hydroxyvitamin D levels.

**Results:**

The study found a high prevalence of 25-hydroxyvitamin D inadequacy, with sufficient levels observed in 65.6% of infants (0–12 months). The lowest prevalence of sufficiency was in the 6–15 years (19.2%) and 16–30 years (22.1%) groups. The highest deficiency prevalence was in the 91–100 years group (51.8%). 25-hydroxyvitamin D levels in all age groups and both sexes correlated with all selected parameters. Lower sun exposure, higher flu virus positivity, male gender, and elevated homocysteine and CRP levels were negatively correlated with 25-hydroxyvitamin D levels.

**Conclusions:**

The prevalence of 25-hydroxyvitamin D inadequacy in the Czech Republic is high. It is advisable to consider testing, monitoring, and providing medical recommendations for vitamin D supplementation as part of health prevention strategies in the general population.

## Introduction

Vitamin D is essential nutrient for maintaining optimal human health throughout whole life. Its deficiency is associated with increased prevalence in osteopenia/osteoporosis, metabolic diseases (metabolic syndrome, obesity, diabetes type II), cardiovascular, neurological (neurodegenerative) and inflammatory diseases and cancer [1–4]. Vitamin D deficiency can be induced by various causes. The most common are decreased exposure to sunlight and inadequate dietary intake of vitamin D. Additionally, lower vitamin D levels can be caused by malabsorption, severe liver and kidney disease, hereditary defects in vitamin D absorption and the use of certain medications such as antiepileptic drugs or corticosteroid treatment [5–7].

The prevalence of vitamin D insufficiency and deficiency in population is very high. Studies have shown that a significant percentage of the global population has inadequate vitamin D levels, even in populations living in warm, sunny climates. Insufficiency and even deficiency were detected in both adults and children [8, 9].

In our study, which is the largest study of 25-hydroxyvitamin D levels in Czech population, we used multidata to determine 25-hydroxyvitamin D levels and the prevalence of low or high 25-hydroxyvitamin D levels in serum in the Czech population. Furthermore, we examined the correlations of 25-hydroxyvitamin D levels with the month of the year, that is, with the period of UV illumination, and the prevalence of influenza infection to determine whether there are fluctuations in 25-hydroxyvitamin D levels and whether these fluctuations are related to these factors.

The data obtained can be used for prevention policy, to evaluate whether 25-hydroxyvitamin D levels in the population need to be monitored, and to recommend supplementation to decision makers to reduce morbidity associated with in adequate levels of 25-hydroxyvitamin D in the population.

## Methods

### Study group, population

This cross-sectional retrospective study was conducted in the Czech Republic, latitude used for analyses 50° 12’ 33.93“N; longitude, 15° 49’ 57.45“E. The study included 119,925 people who visited a general practitioner’s office, medical specialists or (minority) were admitted to hospital. For the analysis, data from the period 2010 to 2020 (prior to covid pandemic) were used.

#### 25-hydroxyvitamin D levels

Levels of 25-hydroxyvitamin D were evaluated by Architect 25-OH Vitamin D with immunoassay analyzer Architect i4000SR and by Alinity 25-OH Vitamin D Reagent Kit (new innovated version of Architect) with immunoassay analyzer ALINITY II (Abbott Laboratories, Abbott Park; USA) according to the manufacturer’s instructions. Detection range of Architect 8.5 to 389.8 nmol/L, ALINITY II 8.8 to 385.5 nmol/L.

#### 25-hydroxyvitamin D status

25-hydroxyvitamin D status was characterized according to the Endocrine Society guidelines for clinical practice [10], which are used in the Czech Republic, including the laboratory that analyzed all blood samples used in the study as follows:

- Normal levels, sufficiency: ≥75 nmol/L
- Lower levels: insufficiency: 50 to <75 nmol/L
- Deficiency <50 nmol/L
- Higher levels: hypervitaminosis: >250 nmol/L

#### Homocysteine levels

Homocysteine levels were evaluated with immunoassay analyzer Architect i4000SR and ALINITY II (Abbott Laboratories, Abbott Park; USA) according to the manufacturer’s instructions (CMIA, chemiluminescent microparticle immunoassay). Detection range 1.0–500 μmol/L.

#### CRP levels

CRP levels were evaluated using AU5800 (Beckman Coulter, Brea; USA) and CRP latex as a reagent with detection range of 0.2–480 mg/L or ALINITY CC (Abott Laboratories, Abbott Park; USA) and CRP vario reagent with detection range of 1.0–480 mg/L according to manufacturer’s instructions (quantitative immunoturbidimetric assay).

#### Sunlight (hours per month)

Average value of sunshine in hours per month were recorded in 2013–2022 in Klementinum, Prague, the oldest weather station in the Czech Republic.

https://www.chmi.cz/historicka-data/pocasi/mesicni-data/mesicni-data-dle-z.-123-1998-Sb#

Sunshine hours are as follows:

January: 40.3, February: 84.4, March: 142.5, April: 184.7, May: 186.6, June: 221.3, July: 228.5, August: 213.9, September: 155.4, October: 112.8, November: 55.5, December: 47.1.

#### Cumulative sunlight

Modeling the Relationship Between Sunlight Exposure and 25-hydroxyvitamin D Levels

It is likely that the 25-hydroxyvitamin D levels change with a slight delay following sunlight exposure since the peak of sunlight occurs in July, while the 25-hydroxyvitamin D levels peak in August. The delayed response is evident as the rise in 25-hydroxyvitamin D after the winter period occurs in May, while the increase in sunlight starts as early as February.

The potential delay in 25-hydroxyvitamin D levels following sunlight exposure can be modeled using a weighted sum of illumination in the preceding months:

1 $$25-{\text{hydroxyvitamin}}\,{\text{D}}\,{\text{level}}\,({\text{month}}) \sim \mathop{\sum }\limits_{\text{year}}\left({\text{Sunlight}}\,{\text{exposure}}\,({\text{month}})\times {\text{Weight}}\,({\text{month}})\right)$$

A simple weight progression is a monotonic decrease in the contribution of sunlight exposure to 25-hydroxyvitamin D levels over time. In this model, the weight of sunlight has the maximum impact in the sample month, and its value decreases towards the preceding months. The dependency is modeled using an exponential curve given by:

2 $${\rm{Weight}}\,({\rm{month}})=\exp \left(\frac{{\rm{month}}}{{\rm{decrease}}}\right)\,{\rm{for}}\; {\rm{month}}=1..12$$

This function is subsequently normalized so that the sum of weights equals one. The steepness of the *decrease* was optimized to achieve maximum correlation between the measured and modeled 25-hydroxyvitamin D data. Optimization was performed using the ‘optimize‘ function in R version 4.3.2 (2023-10-31) (R Core Team (2023). R: A Language and Environment for Statistical Computing. R Foundation for Statistical Computing, Vienna, Austria. <https://www.R-project.org/>.).

#### Flu prevalence

The percentage of flu positive samples in January: 22.1, February: 32.2, March: 23.6, April: 15.5, May: 5.1, June: 0.1, July: 0.0, August: 0.0, September: 0.0, October 1.0, November: 3.5, December: 9.9.

http://atlas.ecdc.europa.eu/public/index.aspx

### Statistics

Statistical analysis was conducted in R version 4.3.2 (2023-10-31) (R Core Team (2023). R: A Language and Environment for Statistical Computing. R Foundation for Statistical Computing, Vienna, Austria. <https://www.R-project.org/>.).

For the assessment of the normal distribution of data, we used the Anderson-Darling test. Since the normality of 25-hydroxyvitamin D was rejected, we reported median, lower and upper quartiles, and minimal and maximal values. For correlation analysis, we used the Spearman test. The level of statistical significance, alpha, was set to 0.05.

## Results

### Demographic data

Data from 119,925 individuals were analyzed in this study. Among participants 75,273 were females and 44,652 were males aged between 0 and 100 years (median 51 and 46 years). Participants were further divided into groups according to age (Table 1).

**Table 1:** Participants according to age (age groups).

Groups (years)	All (*n*)	Females (*n*)	Males (*n*)
0–1	777	325	452
2–5	4066	1722	2344
6–10	5141	2323	2818
11–15	4365	2204	2161
16–20	4168	2485	1683
21–30	9738	6274	3464
31–40	15,390	9876	5514
41–50	17,968	11,422	6546
51–60	17,645	11,713	5932
61–70	21,131	13,994	7187
71–80	14,209	9326	4883
81–90	4999	3450	1549
91–100	328	209	119

*n* number of participants.

### 25-hydroxyvitamin D levels

The levels of 25-hydroxyvitamin D differs according to age. The highest were in children aged 0–1 years (86.60 nmol/L). The lowest in the oldest participants (48.75 nmol/L) (Table 2). There was significant difference between the males and females. Males had lower levels when compared to females (*p* < 0.001) (Table 3).

**Table 2:** Vitamin D levels in each age group.

Groups (years)	All (n)	Q1	Median	Q3	Mean	Min	Max
0–1	777	33.68	86.60	171.24	91.20	6.9	349.7
2–5	4066	28.76	64.50	119.35	66.74	5.5	248.3
6–10	5141	26.55	58.80	110.45	61.25	10.6	305.8
11–15	4365	21.00	52.10	102.60	54.45	8.8	151.5
16–20	4168	18.60	54.80	119.06	58.26	5.5	205.8
21–30	9738	18.10	53.90	119.90	57.38	4.7	400.0
31–40	15,390	18.90	55.30	114.70	57.55	7.5	400.0
41–50	17,968	18.50	56.40	113.88	58.41	1.0	351.4
51–60	17,645	19.10	59.80	115.29	61.27	7.0	208.9
61–70	21,131	18.80	62.10	115.90	62.51	5.1	400.0
71–80	14,209	17.50	62.70	114.40	62.41	5.5	260.1
81–90	4999	14.00	59.40	114.30	59.47	7.5	400.0
91–100	328	10.32	48.75	113.29	51.56	7.5	270.0

*n* number of participants in each group divided by age of participants, *Q1* first quartile, *Q3* third quartile, *Min and Max* minimal and maximal serum 25-hydroxyvitamin D values (nmol/L).

**Table 3:** Vitamin D levels sex differences.

	All (n)	Q1	Median	Q3	Mean	Min	Max	*p* value
Females	75,273	18.88	60.0	117.52	61.59	1.0	400	*p* < 0.001
Males	44,652	18.20	56.4	112.50	58.18	5.3	400	

*n* number of participants in whom levels of homocysteine were measured regardles of age, *Q1* first quartile, *Q3* third quartile, *Min and Max* minimal and maximal serum 25-hydroxyvitamin D values (nmol/L).

### Prevalence of 25-hydroxyvitamin D status; deficiency, insufficiency, sufficiency, hypervitaminosis

The 25-hydroxyvitamin D status was determined in selected age groups. The lowest 25-hydroxyvitamin deficiency and insufficiency was in the infants. 65.6% of infants had sufficient 25-hydroxyvitamin D levels, higher levels were in females (65.8%). The highest prevalence of deficiency was in the 91–100 (51.8%; female 57.9%) but lowest prevalence of sufficiency was in the 6–15 group (19.2%; females 17%). Hypervitaminosis was very rare and occurred more often in females (Table 4).

**Table 4:** The prevalence of vitamin D deficiency, insufficiency and sufficiency in children and adults.

	50 nmol/l deficiency	<50–75 nmol/l insufficiency	<75–250 nmol/l sufficiency	<250 nmol/l
**Children**				
**Group 0–1**				
All *n* = 777	62	204	510	1
Percentage	8	26.3	65.6	0.1
Females				
* n* = 235	33	77	214	1
Percentage	10.2	23.7	65.8	0.3
Males				
* n* = 452	29	127	296	0
Percentage	6.4	28.1	65.5	0
** Group 2–5**				
All *n* = 4066	1016	1741	1309	0
Percentage	25	42.8	32.2	0
Females				
* n* = 1722	450	745	527	0
Percentage	26.1	43.3	30.6	0
Males				
* n* = 2344	566	996	782	0
Percentage	24.1	42.5	33.4	0
** Group 6–15**				
All *n* = 9506	3640	4039	1826	1
Percentage	38.3	42.5	19.2	0
Females				
* n* = 4527	1809	1947	770	1
Percentage	40	43	17	0
Males				
* n* = 4797	1831	2092	1056	0
Percentage	36.8	42	21.2	0
**Adults**				
** Group 16–30**				
All *n* = 13,906	6055	4773	3075	3
Percentage	43.5	34.3	22.1	0
Females				
* n* = 8759	3484	3073	2199	3
Percentage	39.8	35.1	25.1	0
Males				
* n* = 5147	2571	1700	876	0
Percentage	50	33	17	0
** Group 31–50**				
All *n* = 33,354	13,576	12,339	7439	4
Percentage	40.7	37	22.3	0
Females				
* n* = 21,298	8270	7991	5036	1
Percentage	38.8	37.5	23.6	0
Males				
* n* = 12,057	5306	4348	2403	3
Percentage	44	36.1	19.9	0
**Group 51–70**				
All *n* = 38,776	12,857	14,958	10,960	1
Percentage	33.2	38.6	28.3	0
Females				
* n* = 25,657	7949	9881	7826	1
Percentage	31	38.5	30.5	0
Males				
* n* = 22,615	4908	5077	3134	0
Percentage	37.4	38.7%	23.9	0
**Group 71–90**				
All *n* = 19,208	6563	6781	5859	5
Percentage	34.2	35.3	30.5	0
Females				
* n* = 12,776	4034	4423	4315	4
Percentage	31.6%	34.6%	33.8%	0%
Males				
* n* = 6432	2529	2358	1544	1
Percentage	39.3%	36.7%	24%	0%
**Group 91–100**				
All *n* = 328	170	73	84	1
Percentage	51.8%	22.3%	25.6%	0.3%
Females				
* n* = 209	121	43	45	0
Percentage	57.9%	20.6%	21.5%	0%
Males				
* n* = 119	49	30	39	1
Percentage	41.2%	25.2%	32.8%	0.8%

All *n* number of participants in each group, *n females* number of females in the distinct age group, *n males* number of males in the distinct age group, *Percentage* the percentage of participants in whom 25-hydroxyvitamin D deficiency, insufficiency, sufficiency and hypervitaminosis was detected.

### Levels of homocysteine

Levels of homocysteine (µmol/L) were higher in males compared to females (Table 5).

**Table 5:** Homocysteine levels in males and females.

Homocysteine	All (*n*)	Q1	Median	Q3	Mean	Min	Max	*p* value
Male	1453	12.5	15	18.0	15.98	2	106.3	*p* < 0.001
Female	2367	10.7	13	16.1	14.01	1	79.5	

*n* number of participants whose homocysteine levels were measured regardless of age, *Q1* first quartile, *Q3* third quartile, *Min and Max* minimal and maximal serum homocysteine values (mg/L).

### Levels of CRP

Levels of CRP (mg/L) were higher in females compared to males (Table 6).

**Table 6:** CRP levels in males and females.

CRP	All (*n*)	Q1	Median	Q3	Mean	Min	Max	*p* value
Male	33,589	0.8	1.6	3.7	4.21	0	401.0	*p* < 0.001
Female	55,965	0.9	1.9	4.7	4.44	0	330.7	

*n* number of participants whose homocysteine levels were measured regardless of age, *Q1* first quartile, *Q3* third quartile, *Min and Max* minimal and maximal serum CRP values (μmol/L).

### Correlations

We found a strong correlation between 25-hydroxyvitamin D and age, month of the year, sunlight (hours per months in the sampling months), cumulative sunlight (sunlight hour per year) and flu positivity in all participants, females and males.

During the optimization of weighting coefficients, we found the maximum Spearman correlation (rho = 0.3177699) for the decrease (Eq. (2)) of 6.56234. Our optimized model indicates that the contribution of sunlight exposure to 25-hydroxyvitamin D levels decreases with the time elapsed since the sample collection. Assuming that the contribution of sunlight exposure would be greatest at the time of collection, corresponding to 100%, the contributions in the previous eleven months were 85.9%, 73.7%, 63.3%, 54.4%, 46.7%, 40.1%, 34.4%, 29.6%, 25.4%, 21.8%, and 18.7% respectively.

**Fig. 1:**
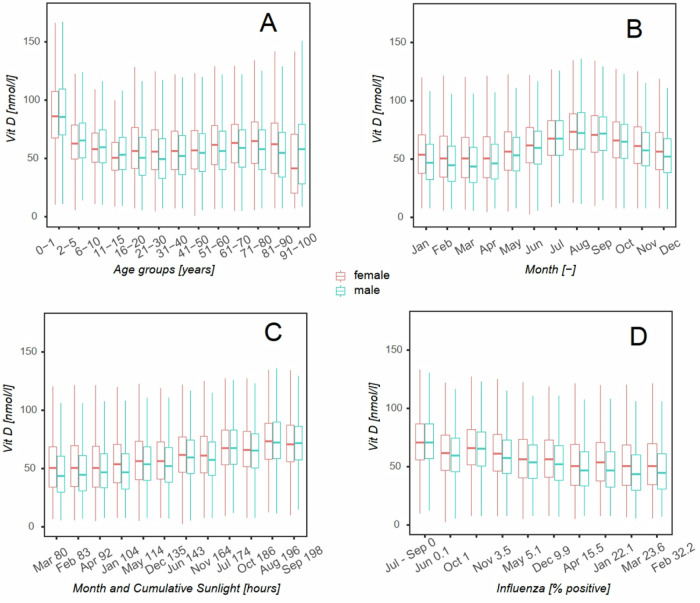
Distribution of vitamin D for male and female subjects stratified by explanatory solid parameters. Data are displayed using box plots (red for female and green for male) delineating the lower quartile, median, and upper quartile, with whiskers indicating the minimum and maximum but no longer than 1.5 interquartile range. Vitamin D levels by age group (years) – **A**; by calendar month – **B**; by cumulative sunlight in one month (hours) – **C**; by influenza prevalence (% positivity) – **D**.

Correlation analysis revealed that 25-hydroxyvitamin D levels are strongly affected by age, sunlight, homocysteine levels and by sex; males are more susceptible to lower levels of 25-hydroxyvitamin D (Table 7, Fig. 1).

**Table 7:** Correlations between vitamin D levels (nmol/L) and selected parameters: age, month of the year, sunlight (hours per months), cumulative sunlight (sunlight hour per year), flu positivity and homocysteine and CRP levels.

Age (years)											
		Q1	Q2	Q3	Mean	SD	Min	Max	Sp	S	*p*-value
All *n*											
Vit D	119,925	41.9	58.7	75.8	60.32	25.65	1	400	0.05	2.74e+14	0
Age	119,925	32.0	50.0	66.0	47.41	22.35	0	100			
Female *n*											
Vit D	75,273	43.1	60	77.3	61.59	25.98	1	400	0.07	6.61e+13	4.25e−82
Age	75,273	35.0	51	66.0	49.24	21.37	0	100			
Male *n*											
Vit D	44,652	40.2	56.4	73.1	58.18	24.93	5.3	400	−0.01	1.5e+13	0.04
Age	44,652	26.0	46.0	64.0	44.34	23.59	0.0	96			

Month of the year											
		Q1	Q2	Q3	Mean	SD	Min	Max	Sp	S	*p*-value
All *n*											
Vit D	119,925	41.9	58.7	75.8	60.32	25.65	1	400	0.21	2.29e+14	0
Month	119,925	3.0	6.0	10.0	6.45	3.52	1	12			
Female *n*											
Vit D	75,273	43.1	60	77.3	61.59	25.98	1	400	0.18	5.82e+13	0
Month	75,273	3.0	6	10.0	6.45	3.51	1	12			
Male *n*											
Vit D	44,652	40.2	56.4	73.1	58.18	24.93	5.3	400	0.25	1.12e+13	0
Month	44,652	3.0	6.0	10.0	6.44	3.54	1.0	12			

Sunglight (hours per month)											
		Q1	Q2	Q3	Mean	SD	Min	Max	Sp	S	*p*-value
All *n*											
Vit D	119,925	41.9	58.7	75.8	60.32	25.65	1.0	400.0	0.13	2.50e+14	0
Sunlight	119,925	55.5	142.5	186.6	134.24	64.66	40.3	228.5			
Female *n*											
Vit D	75,273	43.1	60.0	77.3	61.59	25.98	1.0	400.0	0.11	6.36e+13	2.57e−184
Sunlight	75,273	55.5	142.5	186.6	134.56	64.55	40.3	228.5			
Male *n*											
Vit D	44,652	40.2	56.4	73.1	58.18	24.93	5.3	400.0	0.170	1.23e+13	4.82e−29
Sunlight	44,652	55.5	142.5	186.6	133.69	64.84	40.3	228.5			

Cumulative sunlight (CS; hours per year)											
		Q1	Q2	Q3	Mean	SD	Min	Max	Sp	S	*p*-value
All *n*											
Vit D	119,925	41.9	58.7	75.8	60.32	25.65	1	400.0	0.304	2e+14	0
CS	119,925	71.0	136.7	177.7	136.57	58.07	48	219.8			

Flu (% positive)											
		Q1	Q2	Q3	Mean	SD	Min	Max	Sp	S	*p*-value
All *n*											
Vit D	119,925	41.9	58.7	75.8	60.32	25.65	1	400.0	−0.31	3.77e+14	0
Influenza	119,925	0.1	5.1	22.1	9.80	10.78	0	32.2			
Female *n*											
Vit D	75,273	43.1	60.0	77.3	61.59	25.98	1	400.0	−0.27	9.05e+13	0
Influenza	75,273	0.1	5.1	22.1	9.74	10.79	0	32.2			
Male *n*											
Vit D	44,652	40.2	56.4	73.1	58.18	24.93	5.3	400.0	−0.38	2.04e+13	0
Influenza	44,652	0.1	5.1	22.1	9.90	10.77	0.0	32.2			

Homonocysteine (HMC; µmol/L)											
		Q1	Q2	Q3	Mean	SD	Min	Max	Sp	S	*p*-value
All *n*											
Vit D	119,925	41.9	58.7	75.8	60.32	25.65	1	400.0	−0.07	990	0
HMC	3820	11.3	13.7	16.9	14.76	5.64	1	106.3			
Female *n*											
Vit D	75,273	43.1	60	77.3	61.59	25.98	1	400.0	−0.05	232	<0.05
HMC	2367	10.7	13	16.1	14.01	5.33	1	79.5			
Male *n*											
Vit D	44,652	40.2	56.4	73.1	58.18	24.93	5.3	400.0	−0.06	542	<0.05
HMC	1453	12.5	15.0	18.0	15.98	5.91	2.0	106.3			

CRP (mg/mL)											
	*n*	Q1	Q2	Q3	Mean	SD	Min	Max	Sp	S	*p*-value
All *n*											
Vit D	119,925	41.9	58.7	75.8	60.32	25.65	1	400	−0.04	1.25e+14	0
CRP	89,554	0.8	1.8	4.3	4.36	9.76	0	401			
Female *n*											
Vit D	75,273	43.1	60.0	77.3	61.59	25.98	1	400.0	−0.03	3.01e+13	0
CRP	55,965	0.9	1.9	4.7	4.44	9.26	0	330.7			
Male *n*											
Vit D	44,652	40.2	56.4	73.1	58.18	24.93	5.3	400	−0.07	6.78e+12	0
CRP	33,589	0.8	1.6	3.7	4.21	10.54	0.0	401			

All *n* number of participants regardless of gender and age, *n female* number of females regardless of age, *n males* number of males regardless of age, *Q1* first quartile, *Q3* third quartile, *Min and Max* minimal and maximal values of selected compared parameters, *SD* standard deviation, *S* Spearman rho, S, *p* *p*-value.

The top left graph (A) shows the dependence of 25-hydroxyvitamin D on age. The age ranges for the baskets of this graph are not uniform; their choice was based on the expected dynamics of 25-hydroxyvitamin D change and is described in detail with the numbers of subjects observed in Table 4. The order of the baskets corresponds to the increasing age of the subjects. The top right graph (B) shows the distribution of 25-hydroxyvitamin D levels when the population is divided into baskets according to the month of testing. In this case, each box plot includes persons of various ages. The highest levels were reached in September and August. The bottom left plot (C) represents the same baskets as the top right, but this time, they are ordered by cumulative sunshine duration as calculated by the model for each month - see results for more. The cumulative sunshine data in hours are given in the x-axis description, and the baskets are sorted in ascending order. The plot demonstrates a positive linear dependence on solar cumulative shining, which the correlation analysis (Table 7) confirms. The lower right graph (D) shows the distribution of 25-hydroxyvitamin D levels when the cohort is divided into baskets according to the percentage of influenza detected in independent samples. In this graph, each box plot includes individuals of various ages tested in different months. The percentages of infected samples are given in the x-axis description, and the bins are sorted in ascending order. The plot demonstrates a negative linear relationship confirmed by correlation analysis (Table 7).

**Fig. 2:**
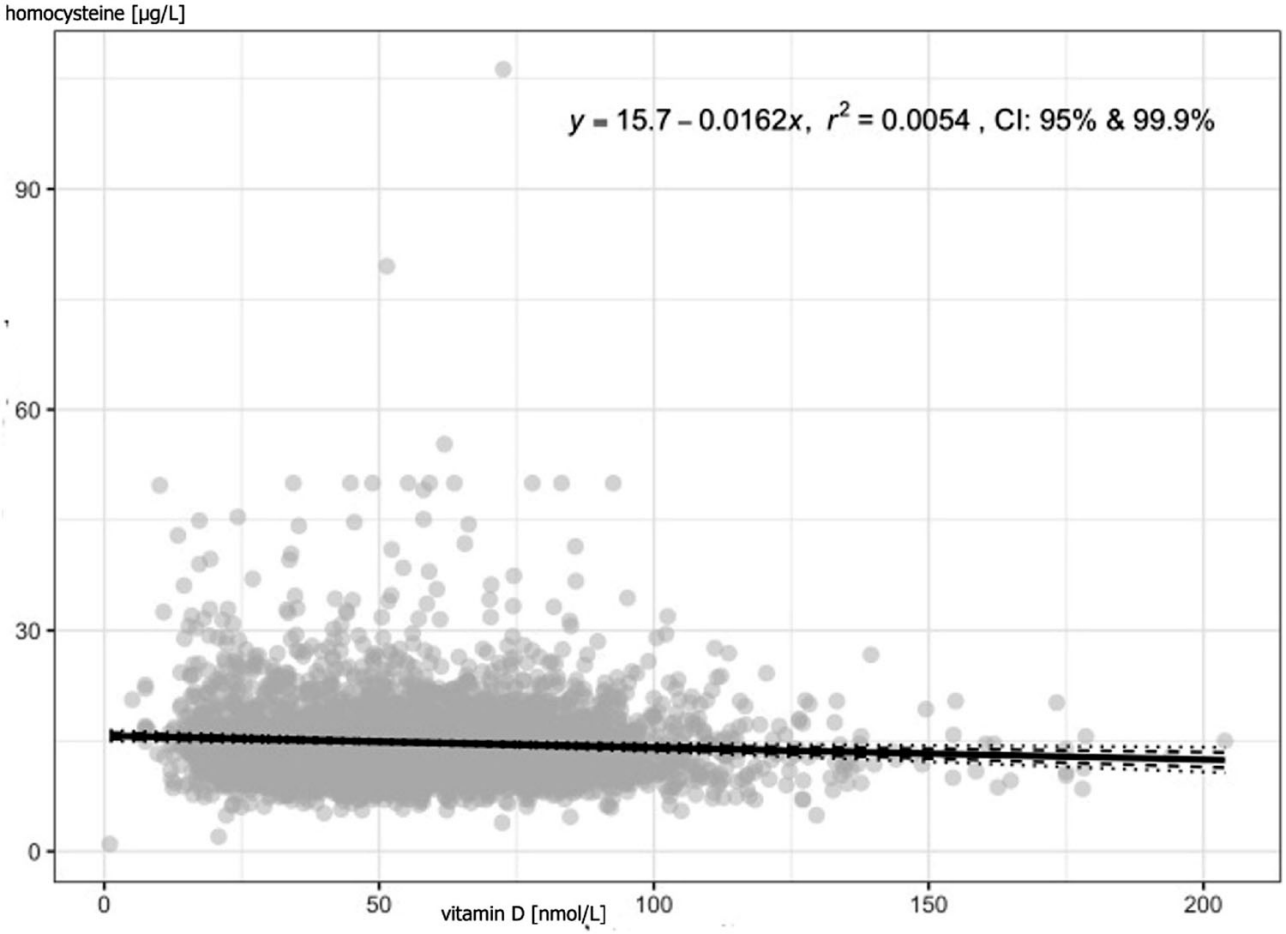
Correlation between vitamin D levels and homocysteine levels in all participants. Each point represents an individual study subject, trendline is the linear black line; r^2^ coefficient of determination.

**Fig. 3:**
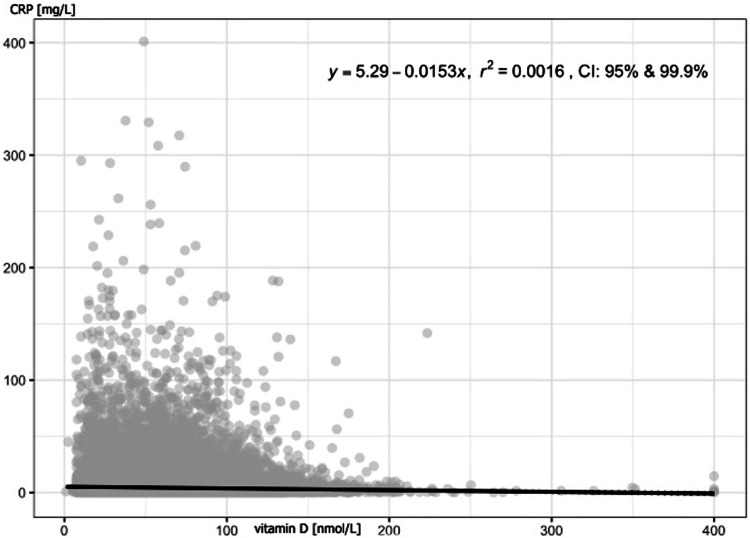
Correlation between vitamin D levels and CRP levels in all participants. Each point represents an individual study subject, trendline is the linear black line; r^2^ coefficient of determination.

Correlation between 25-hydroxyvitamin D levels and CRP was also found (Table 7, Figs. 2, 3; correlation in females and males are in supplementary data).

## Discussion

Adequate amounts of vitamin D are essential for maintaining our health. In our study, we found different 25-hydroxyvitamin D levels in age groups with a high prevalence of insufficiency and deficiency and confirmed the correlation between selected parameters (age, sex, sunlight, flu, and homocysteine).

Currently, there is an ongoing discussion regarding the optimal vitamin D levels necessary for human health. Emerging evidence suggests that a serum 25-hydroxyvitamin D concentration of 50 nmol/L may be fully sufficient, as opposed to the previously recommended level of 75 nmol/L. However, in our study, we adhered to the reference values provided by the laboratory that processed our samples, which are based on the Endocrine Society guidelines used in the Czech Republic. These guidelines define 25-hydroxyvitamin D sufficiency as levels ≥75 nmol/L, insufficiency as 50–75 nmol/L, and deficiency as <50 nmol/L.

To maintain consistency and comparability, our discussion references studies that utilize the same classification ranges. Despite the evolving perspectives on 25-hydroxyvitamin D sufficiency, our findings indicate a high prevalence of 25-hydroxyvitamin D insufficiency and deficiency (levels below 75 nmol/L) across all age groups in the Czech population, while the occurrence of hypervitaminosis was very rare.

Worldwide prevalence of vitamin D deficiency in the total population is high, similar to what we see in our study. Deficiency is present in the population regardless of the location of the country in which the study was conducted. Cui et al. processed data from 308 studies from 2000 to 2022 involving 7.9 million people from 81 countries. 202 studies (7,634,261 participants) showed that the prevalence of 30 nmol/L was 15.7%, 50 nmol/L 47.9% and 75 nmol/L 76.6%. Previous study of Cui et al. focused on the prevalence of vitamin D deficiency in US population (71,685 participants) and described the trends in vitamin D levels from 2001 to 2018. Moderate and severe deficiency was detected in 22.0% and 2.6% (levels 25–50 nmol/L and <25 nmol/L subjects. The prevalence of insufficiency was 40.9% (levels 50–75 nmol/L) while the prevalence of sufficiency was 34.5% [9, 11]. The studies also show that even populations living in sunny areas have problems with inadequate vitamin D levels. Meta-analysis of 96 studies (a total of 227,758 individuals) by Mendes et al. showed that the prevalence of vitamin D deficiency in population of South America is 34.76% [12]. Lebanon study by Harkous et al. evaluated vitamin D deficiency in 66,127 people. The situation in Europe is even worse. Even in just one of the sunniest countries, the prevalence of vitamin D deficits is quite high. Xyda et al. evaluated levels of vitamin D in Greek and Cypriot population. They found that 72.7% (mean level 53.75 nmol/L) of Greek and 69.3% of Cypriot (mean level 64.5 nmol/L) population had inadequate levels of vitamin D [13]. Manios et al. focused on vitamin D status in Southern European and Eastern Mediterranean countries and conducted a systematic review using data from 103 studies with 630,039 subjects. They found that the vitamin D deficiency is, surprisingly, high and suggested that there is the pandemic of vitamin D hypovitaminosis in Europe [14]. The Central Europe and Western Europe has similar problem. Dutch study by Brouwer-Brolsma et al. showed that in 2857 participants aged ≥65 years, the prevalence of vitamin D deficiency (<50 nmol/L) is 45% [15]. In the German study by Rabenberg et al., the prevalence of vitamin D levels <30 nmol/L was 30.2% and the levels >50 nmol/L had 61.6% [16]. The data from Ukraine from 2021 showed that the prevalence of vitamin D deficiency (levels >50 nmol/L) is 26% and insufficiency (50–<75 nmol/L) was 37% [17].

### Levels of vitamin D and prevalence in newborns, infants, and children to 15 years

The youngest participants in our study had the highest levels of vitamin D and the lowest prevalence of vitamin D deficiency (8%). Sufficient vitamin D levels were found in 66% of children aged 0–1 years. We assumed that this result mirrors general acceptance of guidelines of the Czech Pediatric Society (2019) on vitamin D supplementation as a prevention of disorders of bone metabolism, growth, and rickets [18]. Vitamin D supplementation in infants, regardless of the method of nutrition (breastfeeding, formula feeding is recommended in most European countries, including the UK. The study by Uday et al. showed high adherence to recommended vitamin D supplementation across countries [19]. The prevalence of vitamin D deficiency in infants has been documented in countries where there are no guidelines for vitamin D supplementation in children 0–12 months [20, 21].

This positive effect of supplementation continues for the following year of the children’s age. Vitamin D levels, in our study, gradually decreased to the age of 10 years. In children aged 2–5 years the prevalence of sufficiency was 32.2%. The prevalence of deficiency was 25%. In children aged 6–15, the prevalence of sufficiency was only 19.2% and the prevalence of vitamin D deficiency 38.3%.

Similar results were found various studies analyzing the levels of vitamin D in children, e.g. Zhang et al. They evaluated vitamin D status in children (*n* = 6953) aged 0–6 years. In children 0–1 year of age, toddlers and preschool children the median values of vitamin D were 69.4, 62.3, and 50.85 nmol/L, respectively. The levels of vitamin D were seasonally dependent, higher in summer and lower in winter [22]. Vitamin D deficiency and its dependence of season in toddlers confirmed Chaoimh et al. and Stoungjerg et al. The prevalence of deficiency was 26.7% and 38% [23, 24].

As in our study, high vitamin D deficiencies in school children and adolescents have also been reported in many other studies regardless of their origin, including studies from very sunny locations such as Costa Rica, Indonesia and Australia [8, 25–27]. In Chinese study by Hu et al., the prevalence of vitamin D deficiency (lower 50 nmol/L) was 65.98% [28]. On the European continent, studies have been carried out in the Mediterranean area, for example in Italy. Galeazzi et al. measured vitamin D levels in 1706 Italian school-age children. The prevalence of sufficiency (>75 ng/mL) was in 36% while deficiency (25–50 ng/mL) was in 21% and lower levels was in spring and winter [29]. In the Czech Republic, Sochorova et al. showed that in the children aged 5 and 9 (419 subjects) sufficient levels (>75 nmol/L) had only 34% of children. Most children had insufficiency (40%; 50–75 nmol/L). They also found that levels of vitamin D varied according to season. The highest levels were detected in autumn and lowest in spring [30].

### Vitamin D levels in adults and elderly

Our study showed that the levels of 25-hydroxyvitamin D in adults were stable from 15 to 50 years (the prevalence of 25-hydroxyvitamin D deficiency was 16–30 and 31–50 groups (43.5% and 40.7%), then, in older people (51–70 and 71–90 group), we detected the increase in the levels of 25-hydroxyvitamin D and decrease in the prevalence of 25-hydroxyvitamin D deficiency (33.2% and 34.2%) until the age of 90 (51.8%). While the prevalence of 25-hydroxyvitamin D deficiency was higher in younger men compared to women, the opposite was observed in the oldest group. This increase in population aged 50–90 may be due to more frequent preventive examinations in GPs and an increase in morbidity which requires visits to a specialist who can prescribed 25-hydroxyvitamin D supplementation [31–33]. The subsequent decline could be attributed to the fact that such elderly people are often already polymorbid, bedridden and have limited exposure to sunlight. Diet, its composition, quality and ability to absorb nutrients are also problematic. A number of studies evaluated 25-hydroxyvitamin D levels in very old people and centenarians. Kupisz-Urbańska et al. compared the levels of 25-hydroxyvitamin D in adults 65-years old and centenarians. They found that the levels of 25-hydroxyvitamin D in older people were lower, like in our study [34]. Foroni et al. enrolled in their Brazilian study people aged ≥80 years. Severe deficiency (<25 nmol/L) and deficiency (<50 nmol/L) were observed in 13% and 56% subjects [35]. In German study, Rabenberg et al. evaluated levels of 25-hydroxyvitamin D in population 65 to 79 years. The prevalence of deficiency (<30 nmol/L) was 32.9% in females and 26.6% in males. Only 5.7% females and 8.2% males had sufficient (>75 nmol/L) levels of 25-hydroxyvitamin D [36]. Other German study by Conzade et al. confirmed that the prevalence of deficiency is positively associated with age. The prevalence of deficiency (<50 nmol/L) was 44% in the 65–74 age group and increased to 74% in the 85–93 age group [37].

### Correlations

We confirmed that the levels of 25-hydroxyvitamin D depend on the season (months, sunlight, cumulative sunlight), sex, age, incidence of flu, homocysteine, and CRP.

Regarding the seasonal dependence of 25-hydroxyvitamin D levels, our study is consistent with the results of numerous other studies. Some of them were mentioned above. Levels of 25-hydroxyvitamin D decreased during winter and early spring. This effect depends mainly on exposure to sunlight. Australian study by Elliott et al. described that in summer, in all locations, 5–10 min outdoors between 8 am and 4 pm most days of the week, when 35% of the body surface is exposed, is sufficient to maintain current 25(OH)D concentrations. In winter, at mid and higher latitudes, to maintain 25-hydroxyvitamin D concentration, it is necessary to be outdoors in the middle of the day and 10% of the body surface must be exposed for more than 45 min [38].

Study by Fayet-Moore who determined 25-hydroxyvitamin D status in healthy Australian office workers during summer and during winter. 25-hydroxyvitamin D levels were higher in late summer compared to late winter. 25-hydroxyvitamin D deficiency (<50 nmol/L) was detected in 29 (summer) and 42% (winter) subjects [39].

In Slovenian study, by Osredkar et al. found that summer levels of 25-hydroxyvitamin D are significantly higher compared to winter levels, but the levels of 25-hydroxyvitamin D binding protein did not differ [40].

In our study, we also found an interesting association between cumulative annual sunlight and 25-hydroxyvitamin D levels. We found no other study that reports on this. 25-hydroxyvitamin D levels negatively correlated with age which is in accordance with other studies, and male gender, although various studies showed opposite trends [41].

Our results further revealed a correlation between 25-hydroxyvitamin D and the incidence of influenza evidenced by detection of specific antibodies in blood. We have not found a study that confirms a correlation between 25-hydroxyvitamin D levels and influenza, but there are many studies that show that supplementation, i.e., increasing 25-hydroxyvitamin D levels, can reduce the risk of influenza infection. Meta-analysis by Zhu et al. used data from 10 studies with total 4859 participants and showed that supplementation with 25-hydroxyvitamin D can reduce the risk of influenza infections (relative risk 0.78) [42]. A meta-analysis by Martineau et al. confirmed the positive role of 25-hydroxyvitamin D in the prevention of respiratory infectious diseases. They analyzed data from 25 studies with total 11,321 participants aged 0–95 years. In all participants, 25-hydroxyvitamin D supplementation reduced risk of acute respiratory tract infections [43].

We also revealed a negative association between 25-hydroxyvitamin D levels and CRP and homocysteine. Nowadays, the levels of both parameters are frequently assessed, and it has been shown that their increasing values are related to an unhealthy lifestyle and the presence of a number of chronic diseases in which inflammation plays an important role. CRP and homocysteine serve as risk and prognostic factors for cardiovascular, metabolic and neurodegenerative diseases, among others. Similarly, low 25-hydroxyvitamin D levels are associated with the development of these diseases [44, 45].

Zhou et al. conducted the study with data from 294,970 unrelated participants. They confirmed the inverse association between 25-hydroxyvitamin D and CRP in participants with inadequate levels of 25-hydroxyvitamin D and suggested that this association may be caused by 25-hydroxyvitamin D deficiency because 25-hydroxyvitamin D has anti-inflammatory properties [46]. Laird et al. focused on 25-hydroxyvitamin D status and inflammation in the elderly. They measured 25-hydroxyvitamin D and CRP levels in 5381 participants over the age of 50 years. The results showed that participants with sufficient 25-hydroxyvitamin D levels had lower CRP levels. Participants with 25-hydroxyvitamin D deficiency were more likely to have higher levels of CRP [47]. The association between 25-hydroxyvitamin D and homocysteine levels in studies has been investigated among patients suffering from a selected disease, not in the whole population. Verdoia et al. described that patients with cardiovascular disease had lower levels of 25-hydroxyvitamin D, which was also associated with an increase in homocysteine levels [48]. Amer et al. evaluated 25-hydroxyvitamin D and homocysteine levels in 14,630 asymptomatic participants and found an inverse relationship between 25-hydroxyvitamin D and homocysteine in participants with 25-hydroxyvitamin D levels of 21 ng/ml or less [49].

The results of our study suggest that for the Czech population, it would be advisable to set rules for regular 25-hydroxyvitamin D supplementation, especially during late autumn, winter, and early spring.

## Conclusion

This is the largest study of 25-hydroxyvitamin D levels in Czech population. Only limited and earlier studies are available, which do not provide such a comprehensive view of the prevalence of 25-hydroxyvitamin D deficiency in the Czech population. In our study, we confirmed a high prevalence of low 25-hydroxyvitamin D status, which depended on age, sex, sun exposure, prevalence of influenza, CRP and homocysteine levels. The results suggest that a program should be developed to educate the population about the positive impact of 25-hydroxyvitamin D on human health and recommend regular supplementation in doses that ensure adequate 25-hydroxyvitamin D levels.

### Limitations

Although we found correlations between 25-hydroxyvitamin D levels and sunshine hours, we acknowledge that sunshine duration is an imperfect proxy for 25-hydroxyvitamin D synthesis. Factors such as the solar zenith angle—which affects UVB radiation intensity—and individual behaviors like skin exposure, sunscreen use, and lifestyle significantly influence 25-hydroxyvitamin D production. Our reliance on aggregate sunshine hours does not capture these nuances. Future studies should incorporate more precise measures of UVB exposure and consider individual factors. Nevertheless, our findings highlight seasonal variations in 25-hydroxyvitamin D levels and the importance of supplementation during months with minimal endogenous synthesis.

First, as a retrospective study utilizing data over a ten-year period, we were unable to control for potential confounding variables. We lacked detailed individual data on factors influencing 25-hydroxyvitamin D status, such as dietary intake, 25-hydroxyvitamin D supplementation, skin pigmentation, body mass index (BMI), physical activity, clothing habits, time spent outdoors, and sunscreen use, which could substantially impact serum 25-hydroxyvitamin D levels.

Second, despite our large sample size, the study population may not fully represent the entire Czech population. Participants were individuals seeking medical care, potentially introducing selection bias, as they may differ in health status or behaviors from the general population. We also lacked data on socioeconomic status, education level, or other demographic factors that might influence 25-hydroxyvitamin D status.

Third, although we used standardized assays, assay variability exists between laboratories and methods. We did not provide specific data on assay precision and accuracy, nor confirm participation in international standardization programs like DEQAS or VDSP, which may affect comparability with other studies.

Finally, we did not assess the impact of chronic diseases, medications, or genetic factors affecting 25-hydroxyvitamin D metabolism. Conditions such as malabsorption syndromes, liver or kidney disease, and the use of certain medications (e.g., anticonvulsants, corticosteroids) can significantly influence 25-hydroxyvitamin D levels. Not accounting for these factors limits our ability to attribute findings solely to external factors like sunlight exposure.

Despite these limitations, our study offers valuable insights into the prevalence of 25-hydroxyvitamin D deficiency in the Czech Republic, highlighting the need for public health interventions to address this widespread issue.

## Supplementary information

Supplementary information

## Data Availability

The data supporting published results are available from the corresponding author if requested.
