# Supplementary material for: High prevalence of low vitamin D status in the Czech Republic: a retrospective study of 119,925 participants
Source: Eur J Clin Nutr. 2025 Mar 3;79(7):641–52. doi: 10.1038/s41430-025-01587-0 (PMC12274130; doi:10.1038/s41430-025-01587-0)
Supplement: Supplementary file 1 — Supplementary information [file 41430_2025_1587_MOESM1_ESM.docx]

**SUPPLEMENTARY**

**Supplementary table 1. Vitamin D levels according to age in all participants and according to age and sex**

| Females |  |  |  |  |  |  |  |
| --- | --- | --- | --- | --- | --- | --- | --- |
|  | **n** | **Q1** | **Median** | **Q3** | **Mean** | **Min** | **Max** |
| 0–1 | 325 | 33.49 | 86.90 | 171.30 | 90.67 | 10.3 | 349.7 |
| 2–5 | 1722 | 28.11 | 63.20 | 118.99 | 65.84 | 5.5 | 248.3 |
| 6–10 | 2323 | 27.31 | 58.10 | 109.98 | 60.70 | 10.8 | 305.8 |
| 11–15 | 2204 | 20.50 | 50.90 | 97.10 | 53.14 | 8.8 | 151.4 |
| 16–20 | 2485 | 20.21 | 56.70 | 124.14 | 61.17 | 7.5 | 186.0 |
| 21–30 | 6274 | 20.20 | 56.35 | 124.42 | 60.16 | 4.7 | 400.0 |
| 31–40 | 9876 | 19.79 | 56.80 | 117.70 | 59.12 | 7.5 | 385.5 |
| 41–50 | 11422 | 18.80 | 57.30 | 115.50 | 59.13 | 1.0 | 223.4 |
| 51–60 | 11713 | 19.50 | 61.60 | 116.90 | 62.73 | 7.0 | 208.9 |
| 61–70 | 13944 | 19.10 | 63.40 | 116.90 | 63.86 | 5.1 | 400.0 |
| 71–80 | 9326 | 17.10 | 64.90 | 116.80 | 64.29 | 5.5 | 260.1 |
| 81–90 | 3450 | 13.75 | 62.40 | 116.98 | 61.39 | 7.5 | 400.0 |
| 91–100 | 209 | 10.24 | 41.70 | 109.34 | 47.66 | 7.5 | 160.4 |
| Males |  |  |  |  |  |  |  |
|  | **n** | **Q1** | **Median** | **Q3** | **Mean** | **Min** | **Max** |
| 0–1 | 452 | 34.98 | 86.6 | 169.87 | 91.58 | 6.9 | 228.6 |
| 2–5 | 2344 | 29.40 | 65.5 | 119.77 | 67.41 | 14.0 | 194.8 |
| 6–10 | 2818 | 26.40 | 59.5 | 110.66 | 61.69 | 10.6 | 175.4 |
| 11–15 | 2161 | 22.10 | 53.2 | 105.20 | 55.79 | 9.5 | 151.5 |
| 16–20 | 1683 | 17.20 | 50.9 | 109.09 | 53.95 | 5.5 | 205.8 |
| 21–30 | 3464 | 16.00 | 49.5 | 110.64 | 52.35 | 7.5 | 223.4 |
| 31–40 | 5514 | 17.30 | 52.2 | 108.62 | 54.74 | 7.5 | 400.0 |
| 41–50 | 6546 | 17.90 | 55.1 | 110.54 | 57.13 | 5.5 | 351.4 |
| 51–60 | 5932 | 18.60 | 56.4 | 111.34 | 58.37 | 7.5 | 200.0 |
| 61–70 | 7187 | 18.30 | 59.1 | 112.17 | 59.87 | 5.3 | 203.9 |
| 71–80 | 4883 | 18.01 | 58.2 | 109.70 | 58.81 | 7.5 | 156.8 |
| 81–90 | 1549 | 14.67 | 54.7 | 107.06 | 55.21 | 7.5 | 400.0 |
| 91–100 | 119 | 10.40 | 58.1 | 117.84 | 58.42 | 9.1 | 270.0 |

Legend: n, number of participants; Q1, first quartile; Q3, third quartile; Min and Max, minimal and maximal values

**Supplementary table 2. Homocysteine levels according to age in all participants and according to age and sex**

|  | **n** | **Q1** | **Median** | **Q3** | **Mean** | **Min** | **Max** |
| --- | --- | --- | --- | --- | --- | --- | --- |
| 0–1 | 0 | NA | NA | NA | NaN | Inf | -Inf |
| 2–5 | 2 | 7.77 | 09.05 | 10.33 | 09.05 | 7.7 | 10.4 |
| 6–10 | 4 | 6.52 | 07.05 | 11.75 | 8.18 | 6.5 | 12.1 |
| 11–15 | 8 | 6.60 | 10.45 | 16.21 | 10.65 | 6.3 | 17.0 |
| 16–20 | 62 | 6.81 | 12.55 | 22.59 | 13.72 | 6.4 | 44.2 |
| 21–30 | 214 | 7.30 | 12.55 | 25.12 | 13.51 | 2.0 | 50.0 |
| 31–40 | 507 | 6.73 | 12.20 | 23.84 | 12.87 | 3.9 | 36.1 |
| 41–50 | 634 | 7.60 | 12.60 | 23.15 | 13.17 | 1.0 | 33.2 |
| 51–60 | 680 | 7.80 | 13.40 | 25.91 | 14.25 | 5.6 | 106.3 |
| 61–70 | 816 | 8.84 | 13.90 | 27.26 | 14.99 | 6.3 | 79.5 |
| 71–80 | 600 | 9.70 | 15.60 | 30.62 | 16.75 | 8.1 | 50.0 |
| 81–90 | 276 | 10.30 | 17.95 | 34.28 | 19.04 | 9.2 | 45.1 |
| 91–100 | 17 | 12.84 | 22.90 | 39.64 | 23.43 | 12.0 | 40.4 |
| Females |  |  |  |  |  |  |  |
|  | **n** | **Q1** | **Median** | **Q3** | **Mean** | **Min** | **Max** |
| 0–1 | 0 | NA | NA | NA | NaN | Inf | -Inf |
| 2–5 | 1 | 10.40 | 10.40 | 10.40 | 10.40 | 10.4 | 10.4 |
| 6–10 | 1 | 6.50 | 6.50 | 6.50 | 6.50 | 6.5 | 6.5 |
| 11–15 | 3 | 9.39 | 11.10 | 12.43 | 10.97 | 9.3 | 12.5 |
| 16–20 | 31 | 6.47 | 10.90 | 21.65 | 11.93 | 6.4 | 23.9 |
| 21–30 | 127 | 7.13 | 11.40 | 18.38 | 12.19 | 5.6 | 50.0 |
| 31–40 | 323 | 6.10 | 10.90 | 21.57 | 11.73 | 3.9 | 36.1 |
| 41–50 | 366 | 7.30 | 11.50 | 20.26 | 12.06 | 1.0 | 32.7 |
| 51–60 | 391 | 7.50 | 12.30 | 22.32 | 12.99 | 5.6 | 29.0 |
| 61–70 | 536 | 8.70 | 13.50 | 27.88 | 14.69 | 6.3 | 79.5 |
| 71–80 | 396 | 9.50 | 15.20 | 26.71 | 16.21 | 8.1 | 50.0 |
| 81–90 | 180 | 10.20 | 17.85 | 33.27 | 18.62 | 9.2 | 41.0 |
| 91–100 | 12 | 12.71 | 23.30 | 28.12 | 22.92 | 12.0 | 28.2 |
| Males |  |  |  |  |  |  |  |
|  | **n** | **Q1** | **Median** | **Q3** | **Mean** | **Min** | **Max** |
| 0–1 | 0 | NA | NA | NA | NaN | Inf | -Inf |
| 2–5 | 1 | 7.70 | 7.70 | 7.70 | 7.70 | 7.7 | 7.7 |
| 6–10 | 3 | 6.74 | 7.40 | 11.87 | 8.73 | 6.7 | 12.1 |
| 11–15 | 5 | 6.47 | 9.80 | 16.42 | 10.46 | 6.3 | 17.0 |
| 16–20 | 31 | 8.70 | 14.80 | 27.10 | 15.52 | 8.7 | 44.2 |
| 21–30 | 87 | 08.09 | 14.10 | 32.92 | 15.42 | 2.0 | 44.9 |
| 31–40 | 184 | 8.86 | 13.85 | 25.40 | 14.87 | 7.1 | 35.6 |
| 41–50 | 268 | 9.20 | 14.10 | 23.99 | 14.68 | 7.0 | 33.2 |
| 51–60 | 289 | 9.40 | 14.80 | 27.64 | 15.97 | 7.7 | 106.3 |
| 61–70 | 280 | 9.59 | 14.70 | 26.91 | 15.57 | 8.4 | 44.7 |
| 71–80 | 204 | 9.91 | 15.95 | 41.77 | 17.81 | 8.4 | 50.0 |
| 81–90 | 96 | 12.10 | 18.88 | 35.45 | 19.84 | 11.9 | 45.1 |
| 91–100 | 5 | 14.19 | 15.20 | 40.21 | 24.64 | 14.1 | 40.4 |

**Supplementary table 3. CRP levels according to age in all participants and according to age and sex**

| Age groups |  |  |  |  |  |  |  |
| --- | --- | --- | --- | --- | --- | --- | --- |
|  | **N** | **Q1** | **Median** | **Q3** | **Mean** | **Min** | **Max** |
| 0–1 | 388 | 0.1 | 0.4 | 32.32 | 3.10 | 0.0 | 84.00 |
| 2–5 | 2608 | 0.1 | 0.5 | 18.65 | 2.46 | 0.0 | 169.90 |
| 6–10 | 3513 | 0.1 | 0.6 | 15.68 | 2.29 | 0.0 | 107.40 |
| 11–15 | 2860 | 0.1 | 0.5 | 13.30 | 2.00 | 0.0 | 138.00 |
| 16–20 | 2870 | 0.2 | 1.2 | 25.00 | 3.57 | 0.0 | 142.15 |
| 21–30 | 7494 | 0.2 | 1.6 | 23.08 | 04.09 | 0.0 | 188.45 |
| 31–40 | 11877 | 0.2 | 1.6 | 19.91 | 3.70 | 0.0 | 175.10 |
| 41–50 | 14015 | 0.2 | 1.8 | 19.96 | 04.05 | 0.0 | 308.40 |
| 51–60 | 13594 | 0.3 | 2.2 | 19.70 | 4.39 | 0.0 | 256.00 |
| 61–70 | 16001 | 0.4 | 2.5 | 24.00 | 05.05 | 0.0 | 329.20 |
| 71–80 | 10501 | 0.4 | 2.4 | 31.30 | 5.68 | 0.0 | 401.00 |
| 81–90 | 3611 | 0.4 | 2.5 | 45.20 | 6.84 | 0.0 | 330.70 |
| 91–100 | 222 | 0.4 | 2.9 | 94.52 | 11.57 | 0.2 | 218.84 |
| Females |  |  |  |  |  |  |  |
|  | **n** | **Q1** | **Median** | **Q3** | **Mean** | **Min** | **Max** |
| 0–1 | 164 | 0.10 | 0.5 | 34.58 | 3.74 | 0.0 | 56.30 |
| 2–5 | 1103 | 0.10 | 0.7 | 18.24 | 2.49 | 0.0 | 88.80 |
| 6–10 | 1589 | 0.10 | 0.7 | 17.66 | 2.46 | 0.0 | 107.40 |
| 11–15 | 1419 | 0.10 | 0.5 | 12.18 | 1.84 | 0.0 | 138.00 |
| 16–20 | 1685 | 0.20 | 1.5 | 25.72 | 3.82 | 0.0 | 142.15 |
| 21–30 | 4863 | 0.20 | 1.6 | 23.53 | 4.26 | 0.0 | 188.45 |
| 31–40 | 7684 | 0.20 | 1.6 | 20.19 | 3.76 | 0.0 | 175.10 |
| 41–50 | 8814 | 0.20 | 1.8 | 19.87 | 4.12 | 0.0 | 308.40 |
| 51–60 | 8879 | 0.30 | 2.2 | 18.60 | 4.33 | 0.0 | 238.40 |
| 61–70 | 10388 | 0.40 | 2.7 | 23.00 | 05.08 | 0.0 | 329.20 |
| 71–80 | 6768 | 0.40 | 2.5 | 28.38 | 5.42 | 0.0 | 219.40 |
| 81–90 | 2474 | 0.34 | 2.5 | 47.75 | 07.02 | 0.0 | 330.70 |
| 91–100 | 135 | 0.33 | 2.8 | 89.31 | 10.26 | 0.2 | 117.30 |
| Males |  |  |  |  |  |  |  |
|  | **n** | **Q1** | **Median** | **Q3** | **Mean** | **Min** | **Max** |
| 0–1 | 224 | 0.10 | 0.3 | 23.05 | 2.62 | 0.0 | 84.00 |
| 2–5 | 1505 | 0.10 | 0.4 | 18.84 | 2.44 | 0.0 | 169.90 |
| 6–10 | 1924 | 0.10 | 0.5 | 14.19 | 2.15 | 0.0 | 97.90 |
| 11–15 | 1441 | 0.10 | 0.6 | 14.30 | 2.15 | 0.0 | 93.88 |
| 16–20 | 1185 | 0.20 | 1.0 | 23.90 | 3.23 | 0.0 | 92.20 |
| 21–30 | 2631 | 0.20 | 1.6 | 21.90 | 3.77 | 0.0 | 179.92 |
| 31–40 | 4193 | 0.20 | 1.6 | 18.81 | 3.59 | 0.0 | 144.20 |
| 41–50 | 5201 | 0.30 | 1.8 | 20.20 | 3.92 | 0.0 | 187.80 |
| 51–60 | 4715 | 0.40 | 2.1 | 21.20 | 4.50 | 0.0 | 256.00 |
| 61–70 | 5613 | 0.40 | 2.3 | 26.14 | 05.01 | 0.1 | 239.50 |
| 71–80 | 3733 | 0.40 | 2.2 | 39.40 | 6.15 | 0.2 | 401.00 |
| 81–90 | 1137 | 0.40 | 2.5 | 42.55 | 6.43 | 0.2 | 170.40 |
| 91–100 | 87 | 0.52 | 3.3 | 94.12 | 13.59 | 0.3 | 218.84 |

**Correlations between vitamin D and homocysteine levels in females and males**

There is a correlation between vitamin D and homocysteine in both females and males **(**Spearman rho= -0.049; S = 232; p < 0.05 and Spearman rho= -0.06; S = 542; p < 0.05; Fig 1 and 2).

Supplementary Figure 1: Correlation between vitamin D levels and homocysteine levels in females


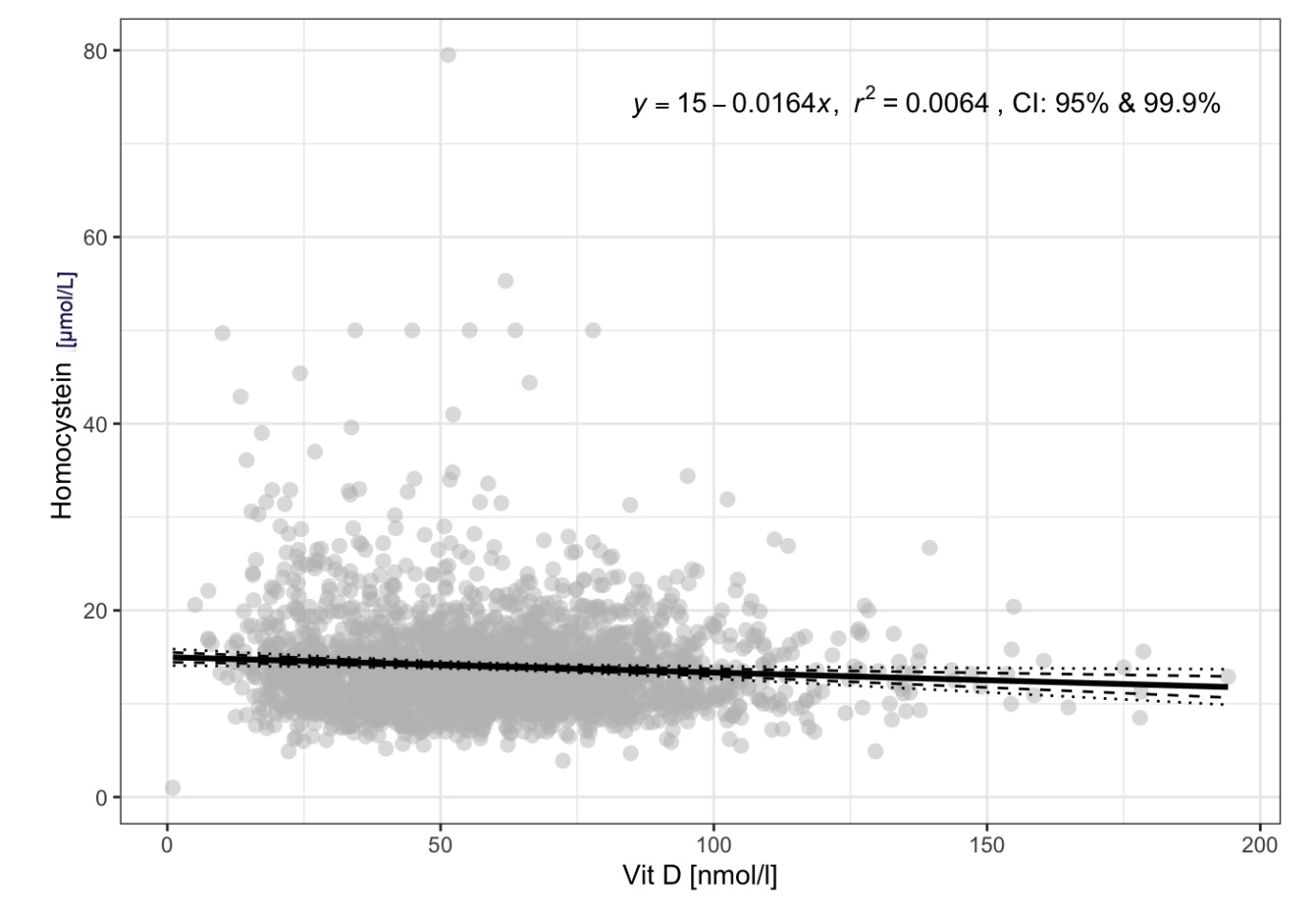


Legend: each point represents an individual study subject, trendline is the linear black line; r^2^, coefficient of determination

Supplementary Figure 2. Correlation between vitamin D levels and homocysteine levels in males


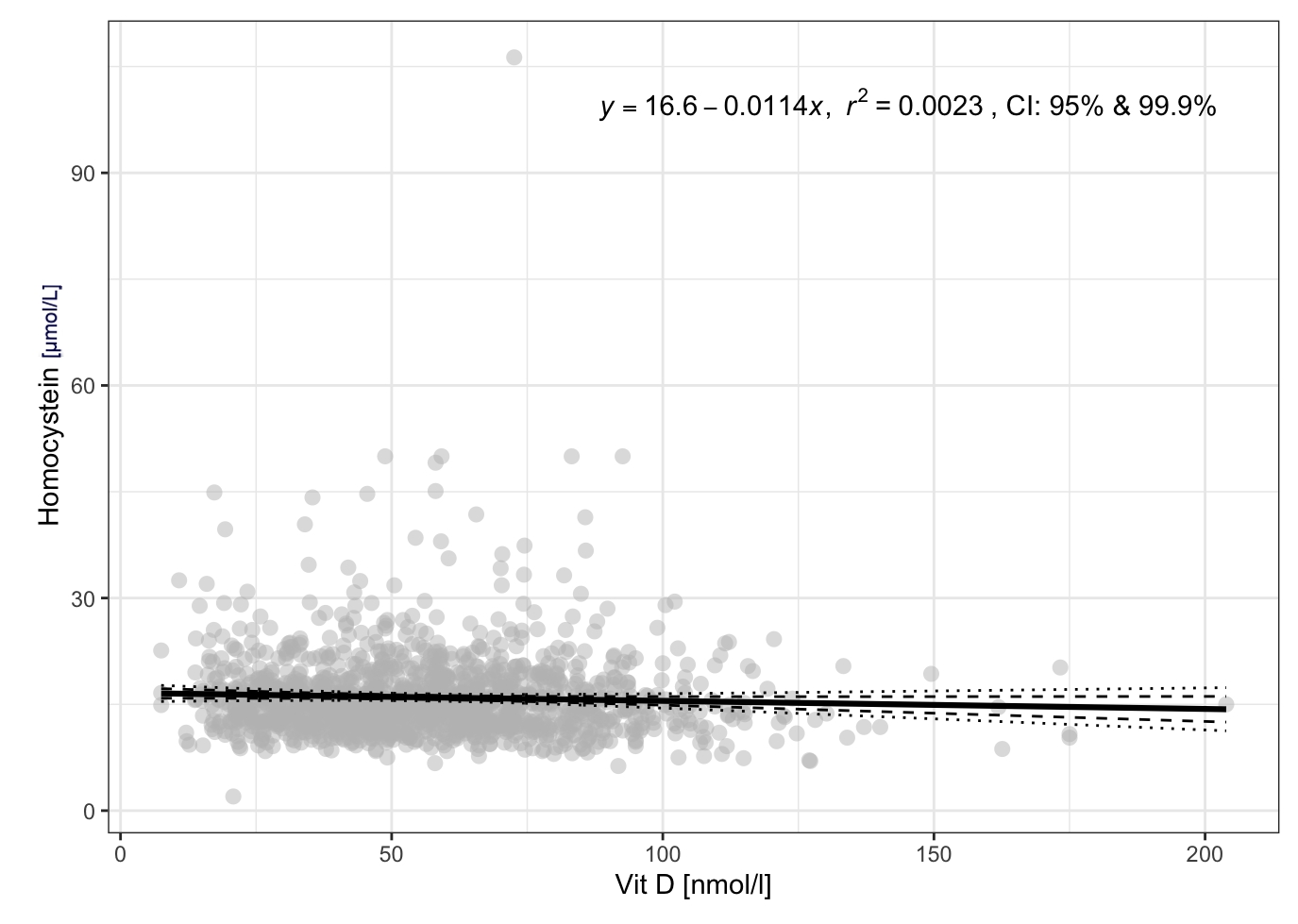


Legend: each point represents an individual study subject, trendline is the linear black line; r^2^, coefficient of determination

There is a correlation between vitamin D and CRP levels in both females and males (Spearman rho= -0.031; S = 3.01e+13; p = 0 and Spearman rho= -0.073; S = 6.78e+12; p = 0; Fig. 3 and 4).

Supplementary Figure 3. Correlation between vitamin D levels and CRP levels in females


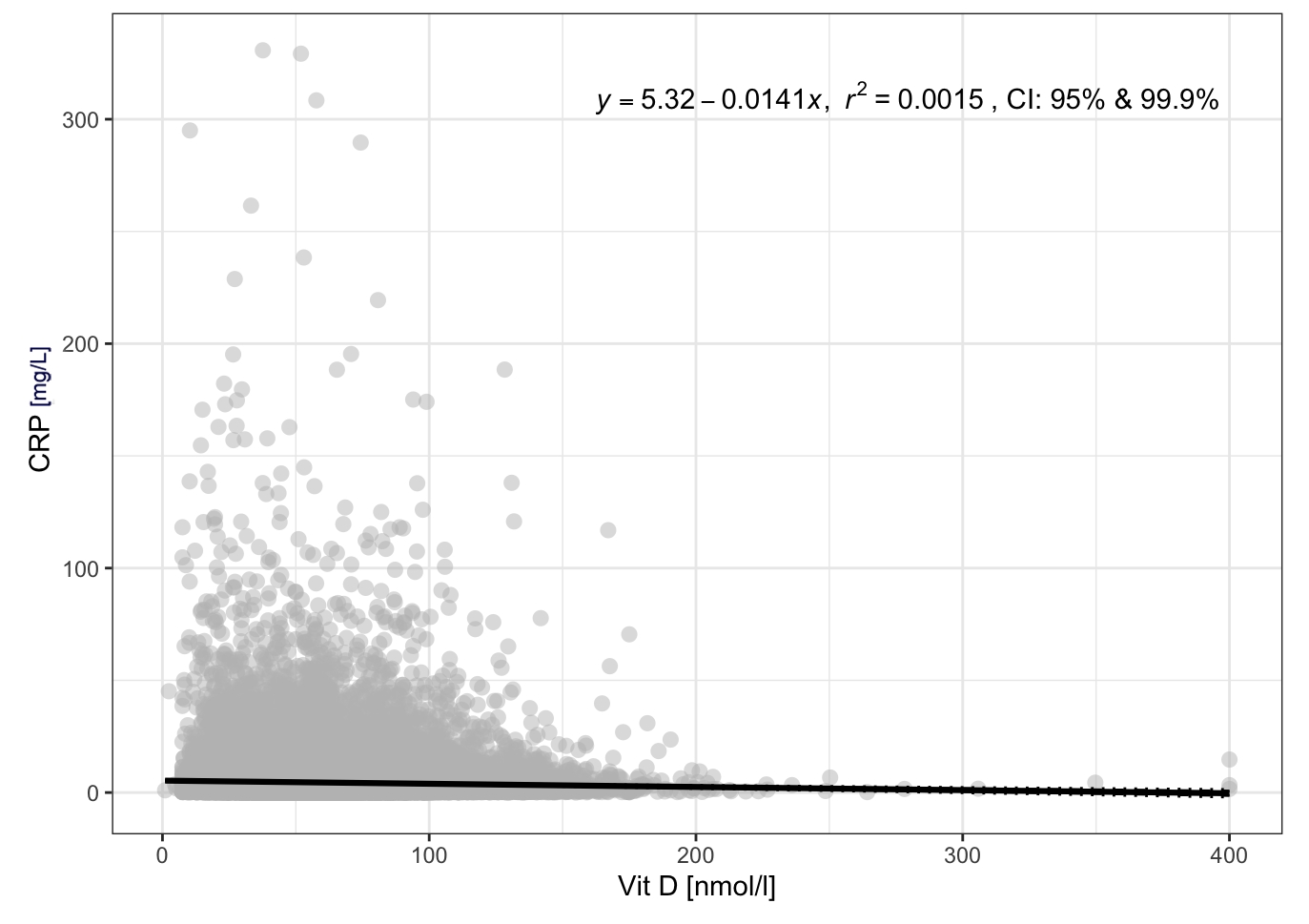


Legend: each point represents an individual study subject, trendline is the linear black line; r^2^, coefficient of determination

Supplementary Figure 4. Correlation between vitamin D levels and CRP levels in males


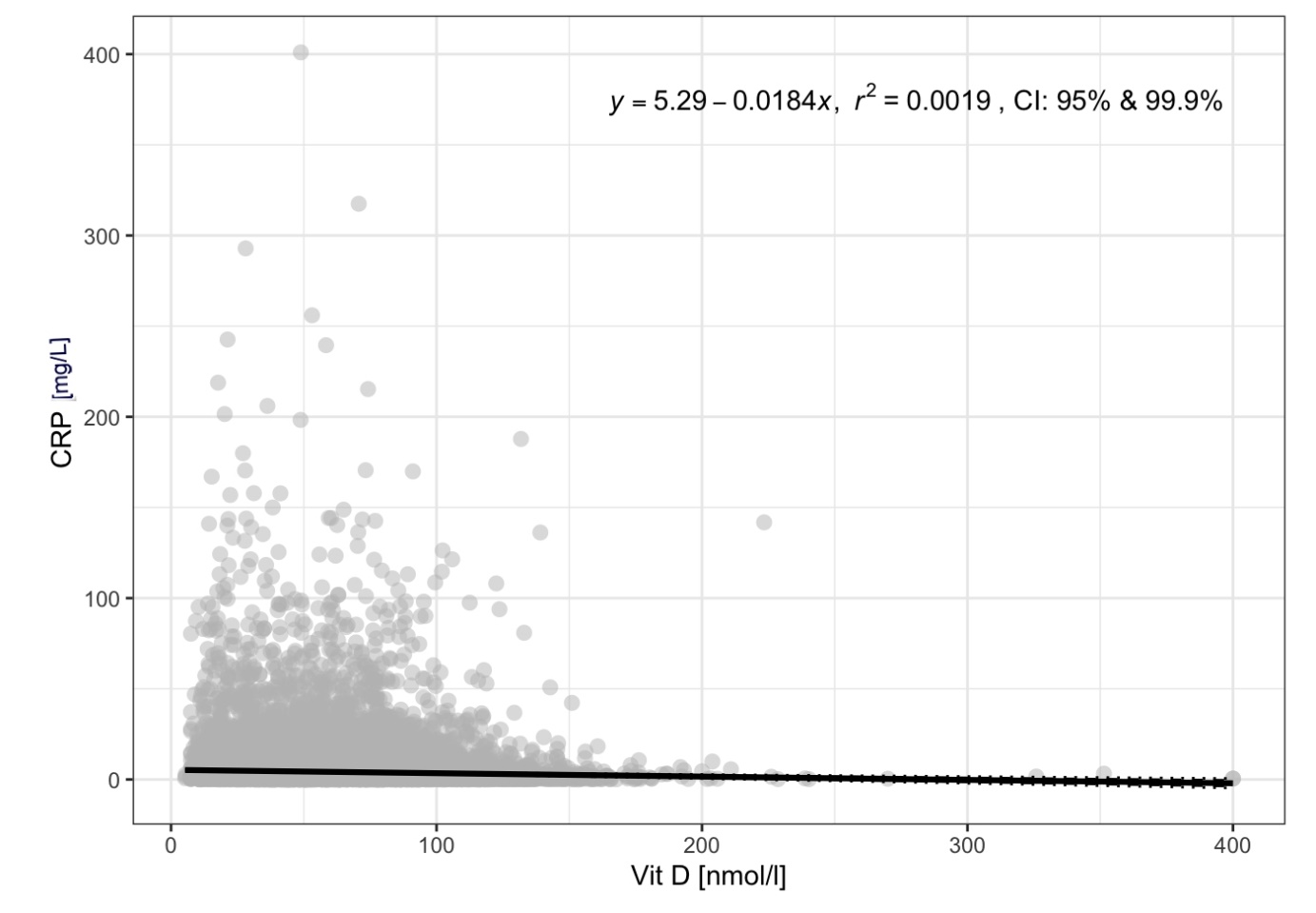
Legend: each point represents an individual study subject, trendline is the linear black line; r^2^, coefficient of determination
